# Convergent Microbial Community Formation in Replicate Anaerobic Reactors Inoculated from Different Sources and Treating Ersatz Crew Waste

**DOI:** 10.3390/life11121374

**Published:** 2021-12-10

**Authors:** Lisa M. Steinberg, Amanda J. Martino, Christopher H. House

**Affiliations:** 1 SciNcite, Newark, DE 19713, USA; 2 Biology Department, School of STEAM, Saint Francis University, Loretto, PA 15940, USA; amartino@francis.edu; 3 Department of Geosciences, Earth and Environmental Systems Institute, College of Earth and Mineral Sciences, The Pennsylvania State University, University Park, State College, PA 16802, USA

**Keywords:** anaerobic digestion, life support system, microbiome, microbial community, amplicon sequencing, next generation sequencing

## Abstract

Future manned space travel will require efficient recycling of nutrients from organic waste back into food production. Microbial systems are a low-energy, efficient means of nutrient recycling, but their use in a life support system requires predictability and reproducibility in community formation and reactor performance. To assess the reproducibility of microbial community formation in fixed-film reactors, we inoculated replicate anaerobic reactors from two methanogenic inocula: a lab-scale fixed-film, plug-flow anaerobic reactor and an acidic transitional fen. Reactors were operated under identical conditions, and we assessed reactor performance and used 16s rDNA amplicon sequencing to determine microbial community formation. Reactor microbial communities were dominated by similar groups, but differences in community membership persisted in reactors inoculated from different sources. Reactor performance overlapped, suggesting a convergence of both reactor communities and organic matter mineralization. The results of this study suggest an optimized microbial community could be preserved and used to start new, or restart failed, anaerobic reactors in a life support system with predictable reactor performance.

## 1. Introduction

Future projects for NASA and private space agencies include the establishment of an orbiting Moon base as well as manned travel to Mars. The high costs and size constraints associated with shipping food makes this scenario unlikely to support long-term manned space travel. Therefore, future manned space operations will require efficient recycling of water and nutrients from organic waste for reincorporation back into food production. Currently, on the International Space Station, fecal waste is collected and stabilized for storage prior to shipment back to Earth for disposal [1]. Urine is collected separately from fecal waste with water recovered by vapor compression distillation and the concomitant production of a nutrient-rich brine [1]. In both scenarios, valuable nutrients are not reused, but rather collected for disposal. There are currently no waste treatment systems validated for space travel that are able to recover nutrients from food and metabolic wastes for reincorporation into food production.

Due to the high water content of urine, feces, and food waste, thermal processes such as combustion, pyrolysis, or gasification would be energy-intensive, and therefore, undesirable for incorporation into life support systems [2]. Microbial waste treatment is a better option as this is capable of mineralizing a wide variety of wastes while yielding valuable nitrogen and phosphorus nutrients for crop production. Both aerobic and anaerobic microbial treatment systems have been investigated for nutrient recycling in space operations [3,4], although anaerobic systems are preferable as these do not require expensive aeration and produce less microbial biomass. Some studies examining anaerobic waste treatment for space travel have focused on the suppression of methanogenesis to produce a high VFA, high ammonia effluent [5,6]. However, completely preventing methanogenesis is operationally challenging, and methanogenic anaerobic treatment is capable of mineralizing the bulk of organic carbon to CO_2_ and methane. The separation of these gases produces a concentrated CO_2_ stream that can be used for the growth of algae as a food source [7,8], and the recovered methane can be used for energy production or supplemental food production [9]. Despite concerns about the use of flammable gases during space travel, methane is currently part of atmosphere regeneration on the ISS [10], and long-term mission strategies propose to continue this practice for atmosphere regeneration [11]. The current use of methane gas during the operation of the ISS suggests methane can be safely controlled and utilized in future space missions. Therefore, anaerobic waste treatment is a viable option for recycling nutrients in life support systems.

The anaerobic breakdown of organic matter follows sequential steps mediated by a consortium of microbes [12]. The first step is the hydrolysis of the polymeric substances (polysaccharides, lipids, proteins) into smaller substrates (sugars, fatty acids, amino acids). These are, in turn, utilized during the next stage in the process, termed acidogenesis, in which fermentation produces primarily volatile fatty acids (VFAs) and alcohols, CO_2_, and hydrogen, along with ammonia and sulfide. The final stages, acetogenesis and methanogenesis, overlap. Methane is produced by methanogens through three main pathways. Hydrogenotrophic methanogenesis reduces CO_2_ to methane with electrons from H_2_, acetotrophic methanogenesis ferments acetate to CO_2_ and methane, and methylotrophic methanogenesis produces methane from methylated substrates such as methanol and methyl sulfides. Acetogenesis converts the fatty acids and alcohols produced during acidogenesis to acetate, H_2_, and CO_2_ in syntrophic association with other members of the consortium, often methanogens [12]. 

Anaerobic digestion technology has been widely deployed to treat a variety of organic wastes including municipal wastewater sludge, animal manure, agricultural residues, and food waste. Some researchers have attempted to identify a “core microbiome” necessary for anaerobic digestion, yet there is minimal consensus in these findings. Over the course of a year, Calusinka et al. monitored 20 mesophilic, full-scale, continuously stirred tank reactors (CSTRs) treating either municipal wastewater sludge, animal manure, or a combination of the organic fraction of municipal solid waste and agricultural waste [13]. The microbiomes of individual reactors varied little over the monitoring period. Core members of the *Archaea* present at all sampling points represented 12.4% of OTUs but 75.3% of sequence abundance. Similarly, core bacteria represented 2.5% of OTUs but accounted for 70.3% of reads, suggesting that microbes representing a rather limited number of OTUs performed the bulk of anaerobic digestion. Thirteen phyla were present in all the samples, including *Euryarchaeota*, *Firmicutes*, *Bacteroidetes*, *Cloacimonetes*, *Proteobacteria*, *Tenericutes*, *Spirochaetes*, *Aminicenantes*, *Chloroflexi*, and *Parcubacteria*. Hassa et al. examined the microbiomes of 67 CSTRs at 49 different biogas plants differing in operational temperature, retention time, and feedstocks [14]. A few dominant phyla (e.g., *Euryarchaeota*, *Firmicutes*, *Bacteroidetes*, *Cloacimonetes*, and *Tenericutes*) were similar to those reported by Calusinska et al. [13]. However, *Chloroflexi*, *Spirochaetes*, *Aminicenantes*, *Parcubacteria*, and *Proteobacteria* were not detected in all the reactors. Additionally, the researchers detected the predominance of different phyla, specifically *Actinobacteria* and *Atribacteria* [14], suggesting that these phyla could be considered part of the anaerobic digestion core microbiome. Sundberg et al. determined the microbial communities of 21 full-scale CSTRs, operated at mesophilic or thermophilic conditions, and fed either municipal wastewater sludge, or mixtures of co-digested substrates including slaughterhouse waste, household and restaurant food waste, agricultural residues, and manure [15]. Sequences belonging to *Firmicutes* (classes *Clostridia*, *Bacilli*, and *Erysipelotrichia*) and *Bacteroidota* (classes *Sphingobacteria* and *Bacteroidetes*) were the only bacterial phyla detected in all the reactors [15].

These studies suggest a fairly wide diversity of digester microbiomes can function to break down organic matter and recycle nutrients in a life support setting. This functional redundancy is useful in preventing digester upsets; however, it may also cause a lack of predictability in the responsible microbial systems. Ideally, the resultant microbial community and concomitant performance of a digester should be predictable based upon the parameters that can be controlled in the life support system. Development of the microbial community in anaerobic reactors is attributed to both stochastic and deterministic processes. Stochastic processes result from random assembly of the microbial community due, in part, to the potential for functional redundancy in the system. Deterministic processes include the operational control of parameters, such as inoculum, temperature, substrate, organic loading rate, retention time, and reactor configuration. The aforementioned surveys of full-scale CSTRs determined that digester microbiomes are clustered by both temperature [14,15] and substrate [13,15], suggesting performance and microbial communities can be predicted by operational parameters. However, in these studies, reactors were receiving unsterilized waste and so there could be some contribution from the substrate to the microbiome. Han et al. sought to determine the role of the inoculum in the resultant digester microbiome by inoculating CSTRs from full-scale reactors (CSTRs and granular), as well as natural (manure) sources [16]. Reactors were fed a sterilized basal medium containing cellulose that prohibited reactor colonization from the substrate. The sequencing of reactor communities and inoculum sources showed a clustering of each reactor microbial community with its inoculum source, suggesting a strong influence of the inoculum on the resultant reactor community. Despite developing different communities, the reactors inoculated from different sources demonstrated similar performance, suggesting functional redundancy. However, the triplicate reactors inoculated from a single source demonstrated different performance and different microbial communities, also suggesting stochastic influences in community assembly [16]. Lemoine et al. inoculated replicate batch reactors from three full-scale digesters, two granular and one CSTR, which were fed pasteurized synthetic dairy wastewater until stable operation was achieved [17]. Performance was similar for all the reactors, regardless of the inoculum. The sequencing of the microbial communities of these reactors showed only about 25% of OTUs were shared among all the reactors. However, these shared OTUs accounted for over 98% of sequence reads, suggesting similar dominant communities in all the reactors. Similar to the findings of the other studies, the samples in this study clustered by the reactor from which they were taken, and the replicate reactors clustered according to the inoculum source, suggesting persistent and detectable differences in the community structure. Additionally, microbial communities in the lab-scale reactors were more similar to each other than were the communities of the original inocula, suggesting some community convergence in the lab-scale reactor environment [17]. Community convergence was also detected by Peces et al., who examined the temporal community changes of CSTRs processing cellulose and casein that were inoculated from four full-scale anaerobic reactor sources [18]. Over time, the reactors converged in both chemical profile and microbial community, with 52% of the identified OTUs shared among all the digesters, representing 72% of the relative abundance. The authors concluded deterministic rather than stochastic processes were important in determining microbial communities [18].

These studies suggest that although the inoculum source results in detectable differences in the resultant communities, functional redundancy can result in effective and reproducible performance with different communities. These findings are important in the context of a life support system where a reactor may need to be reinoculated from a preserved inoculum, or even from the waste it is treating. Additionally, the choice of reactor type is important for predicting its performance and microbial community. In the previously mentioned studies, reactors were operated as either CSTRs or batch reactors. The small confines of a life support system will require an efficient, high-rate processing of organic waste, which is better suited to a fixed-film reactor. The anaerobic microbial breakdown of organic matter generally proceeds more slowly than aerobic breakdown, as less energy is available from anaerobic processes. However, the use of fixed-film reactors can overcome this limitation by separating the hydraulic retention time of the waste from the solid retention time of the anaerobic microbes, allowing rapid and nearly complete organic matter mineralization with rather compact reactor sizes [19]. Additionally, fixed-film anaerobic reactors are resistant to changes in the organic loading rate [20], toxins [21], high ammonia concentrations [20,22], and even intermittent operation [23], thereby reducing operational complexity. More recently, developments in anaerobic membrane reactors have shown this technology to be capable of low energy, high-rate organic matter breakdown with the production of a nutrient-rich, microbe-free effluent that can be subsequently used for crop cultivation or further refined to potable water [3,24]. The initial testing of an anaerobic membrane reactor for inclusion in future manned space missions has yielded promising results, including the removal of 99% of volatile solids and resistance to shut-down periods of up to 147 days [3]. However, few studies have examined the reproducibility of performance and microbial community in fixed-film reactors inoculated from different sources. This is likely due, in part, to the predominance of CSTRs in full-scale digester operations. Cheng et al. examined microbial communities of lab-scale packed-bed membrane reactors and identified core genera present in more than 90% of reactor samples [25]. These genera represented between 2.4 and 2.9% of sequence reads, and largely belonged to methanogens and their syntrophic fermentative partners. These findings suggest the fixed-film reactor environment selected for a small portion of the community with stochastic assembly describing the bulk of the microbial community [25].

The most important factors for the deployment of microbial systems in space operations are reliability and predictability. Given the operational efficiency and durability of fixed-film and/or membrane reactors, these are likely candidates for inclusion in a life support system. However, there is a lack of studies examining the reproducibility of microbial community formation in fixed-film reactors, from either naturally occurring or constructed reactor inocula. Therefore, the purpose of the current study was to examine the reproducibility of microbial community development in fixed-film anaerobic digesters inoculated from natural and constructed methanogenic environments and operated under identical conditions. The purpose of this work was to elucidate the influence of both the inoculum and the reactor environment in providing predictable, reproducible microbial communities and performance in replicate reactors inoculated from the same source, as well as the degree to which functional and phylogenetic convergence can occur from diverse microbial inocula.

## 2. Materials and Methods

### 2.1. Construction and Operation of Fixed-Film, Flow-Through Anaerobic Digesters

**Figure 1:**
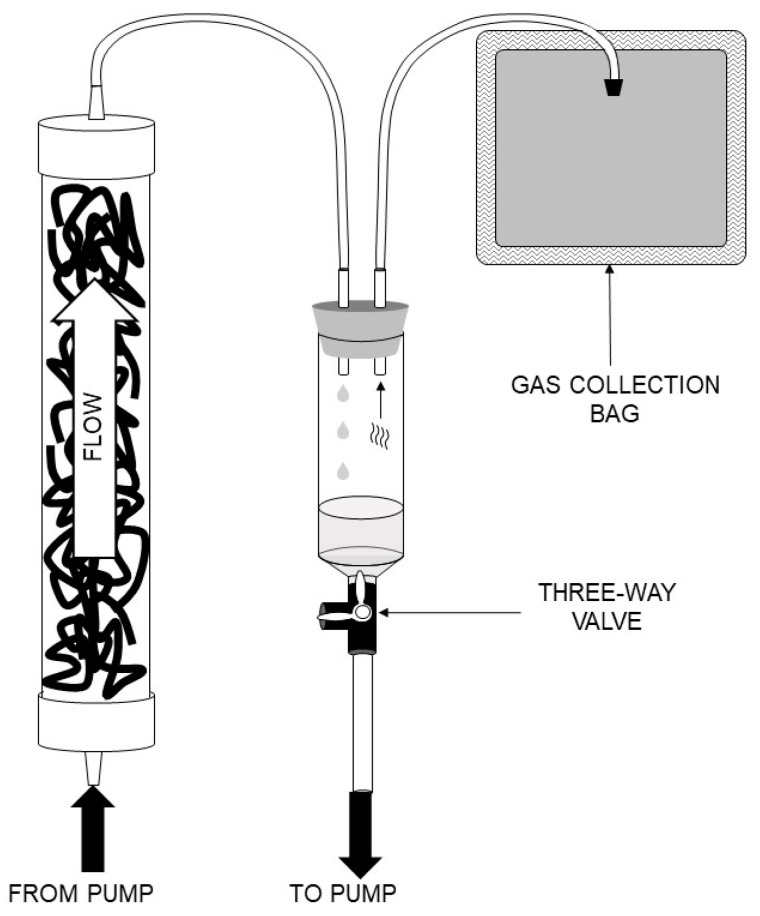
General schematic diagram for construction and operation of small anaerobic reactors. Reactors were operated in an upflow configuration with subsequent liquid/gas separation in a syringe. Gas was passively collected in a gas collection bag. Effluent was recirculated back to the influent via a peristaltic pump. A three-way valve at the bottom of the syringe allowed feeding and removal of effluent from the reactor.

In a previous study, a 12-L, fixed-film, plug-flow anaerobic reactor was constructed and tested for potential inclusion in a life support system for waste treatment and nutrient recycling [9]. Smaller-sized, fixed-film, plug-flow anaerobic reactors were constructed to test the reproducibility of biofilm formation from the same inoculum. These small-scale reactors were constructed in a similar manner to the large reactor, using threaded cPVC tubing with dimensions of 12 in (25.4 cm) in length and 1.5 in (3.8 cm) in diameter. Figure 1 provides a general schematic diagram for construction and operation of a small reactor with detailed construction information provided in the Supplementary Methods. The reactors were connected to a peristaltic pump fitted with a multichannel pump head via Tygon Masterflex B-44-4X tubing (Saint-Gobain, Akron, OH, USA) that possesses low gas permeability. The reactors were packed with 20 g of Bacti-Twist plastic filter media (Creative Products & Services, Inc., Frankfort, KY, USA), a heterogeneous mix of plastic strips of varying widths and lengths. The packed reactor volume was 600 mL. All tubing and fittings that could be autoclaved were sterilized in this manner, and all other fittings were thoroughly cleaned with soap and water, then soaked for at least one hour in 70% ethanol. After all reactor parts were cleaned and sterilized, the reactors were assembled, and a sterile 70% ethanol mix was pumped through the reactors for 24 h. The reactors were drained and allowed to dry before inoculation.

A total of eight small reactors were constructed and operated. Two control reactors (Ctl 1–2) were uninoculated and fed only sterile medium. The anaerobic medium used for inoculation and subsequent feeding was the same as that used to support the large anaerobic reactor and was adapted from recipes for ersatz black water [26]. This medium represented a synthetic wastewater with approximately 18% total solids, 12% volatile solids, and 21 g/L COD. The medium was prepared under anaerobic conditions and dispensed in glass serum bottles (Wheaton Industries, Millville, NJ, USA) sealed with blue chlorobutyl rubber stoppers (Bellco Glass, Inc., Vineland, NJ, USA) and crimped aluminum caps, and autoclaved to sterilize. Two reactors (Bog 1–2) were inoculated with peat from an acidic transitional fen, Bear Meadows Natural Area, which sits in Rothrock State Forest in central Pennsylvania, USA. Bear Meadows Natural Area is a peat bog dominated by *Sphagnum* mosses and sedges with some highbush blueberry [27]. Water input to the area is predominately rainwater supplemented by groundwater seepage with peat over a meter in depth and an average pH of 4.5. This peatland is known to host a robust methanogenic community. Peat was harvested from approximately 20 cm below the water surface and transported and processed under anaerobic conditions. In order to use the peat as a reactor inoculum, the peat was vigorously mixed in sterile, anaerobic medium for one hour to break up the peat and release microbes into solution. Large solids settled out of the solution, and the remaining liquid was used to inoculate the reactors. The remaining four reactors (AD 1–4) were inoculated from the large anaerobic reactor, which, at that point, had been in operation for 445 d. Twenty Biofilter balls were removed from the large reactor and transferred into sterile, anaerobic medium and stirred vigorously for one hour to dislodge the attached biomass that was then used to inoculate the small reactors.

Reactors were operated in an upflow configuration with a continuous recirculation of effluent to influent via a peristaltic pump equipped with a multi-channel pump head operated at a flow rate of 5 mL/min. The effluent exited the reactors at the top where it dripped into a sealed 60-milliliter syringe fitted with a #5 neoprene stopper cored with two ¼ in (0.64 cm) holes. One hole accepted the effluent from the top of the reactor and the other hole accepted tubing, which led to a 1-L Cali-5 Bond bag (Calibrated Instruments, Inc., McHenry, MD, USA) that has very low permeability for methane and hydrogen gases. The areas around the tubing were sealed with silicone sealant to prevent gas leakage. Fluid levels in the system were adjusted so that the syringe barrel contained approximately 10 mL of fluid, thereby allowing gas evolution and passive filling of the Cali-5 Bond bag. A three-way Luer-lok valve located at the base of the reactor allowed feeding via an attached syringe. A similar valve located at the base of the syringe receiving reactor effluent allowed withdrawal of effluent from the reactor. To minimize oxygen introduction, syringes for feeding and effluent collection were equipped with valves, and gas sampling bags were flushed with N_2_, then vacuumed empty prior to reattaching these to the reactor system. After inoculation, the small reactors were operated in continuous recirculation with routine gas collection and measurement, effluent withdrawal, and feeding. Unlike the large reactor, the small reactors were unheated and maintained at the temperature of the laboratory (average 21–22 °C). For the first month of operation, the reactors were fed 20 mL of sterilized, anaerobic medium once per week with 20 mL of effluent subsequently withdrawn. For the second month of operation, feeding and effluent withdrawal of 20 mL was performed two times a week, and thereafter it was performed three times a week. This schedule resulted in a final hydraulic retention time of 70 d and a mass loading rate of 0.3 kg·m^−3^ d^−1^.

### 2.2. Analytical Chemistry

Evolved reactor gas was collected in Cali-5 Bond bags for determination of gas volume and composition. Gas volume was measured daily, and gas composition was measured three times per week. Gas volume production was measured by drawing gas from the bags with an air-tight graduated syringe. Gas composition was determined via gas chromatography with a model 8610C GC (SRI Instruments, Torrance, CA, USA) equipped with a 6-foot (183 cm) MS13X molesieve column (Restek, Bellefonte, PA, USA) and a helium ionization detector at a constant pressure of 23 psi. The temperature program included an initial temperature at 40 °C for 4 min 30 sec followed by a 40 °C/min ramp to 220 °C, which was held for 3 min 30 s. Laboratory grade gases of methane, hydrogen, nitrogen, and carbon dioxide were used for external calibration. Effluent pH was determined via a pH meter equipped with a Ag/AgCl probe. The COD of effluent from the reactors was determined with Chemetrics (Midland, VA, USA) HR+ COD vials with a measurement range of 0–15,000 ppm using a COD standard (Chemetrics, Midland, VA, USA). Volatile fatty acids (VFAs) were quantified on a Dionex LC30 equipped with an IonPac AS15 anion exchange column operated at 31 °C. Anions were eluted with a gradient of potassium hydroxide from 2–25 mM over 20 min at a 1 mL/min flow rate with ECD and suppressor currents at 97 and 180 mA, respectively. A standard free VFA (Sigma-Aldrich) was used for external calibration.

### 2.3. Reactor Microbial Ecology

Plastic media were removed from the small reactors for biomass recovery and DNA extraction on day 307 of operation. Approximately 1 g of plastic media was taken from the bottom (inflow) and top (outflow) of each reactor, and 5 mL of recirculated fluid (effluent) was extracted from the sampling syringe for each reactor. Plastic media were placed into a 50-milliliter conical centrifuge tube containing 25 mL of 0.1 M Tris buffer, pH 8.0, with 0.05 M NaCl, 0.2% Na-pyrophosphate, and 0.1% Triton-X 100. Tubes were vortexed at maximum speed for 10 min to dislodge the biofilm from the plastic media. Cells were collected by centrifugation and resuspended in 2 mL of 10 mM Tris buffer, pH 7.5. DNA was extracted with a MoBio Power Soil DNA extraction kit (Mo Bio Laboratories, Carlsbad, CA, USA). Triplicate DNA extractions were performed per reactor and location (Top, Bottom, and Effluent), with these triplicate extractions pooled prior to analysis. Amplicons for 454 FLX sequencing were obtained with Pfu Ultra HF DNA polymerase (Stratagene, La Jolla, CA, USA) using previously developed primers [28], which target the V6-V7 region of the 16S gene, and included 454 Titanium adapter sequences and pre-programmed bar codes (Table S1). The amplification protocol included initial denaturation for 5 min at 94 °C followed by 30 rounds of amplification with denaturation at 94 °C for 30 s, annealing for 20 s at 53 °C, and elongation at 72 °C for 1 min, with a final elongation step of 10 min at 72 °C. PCR products were purified from agarose gel and eluted in PCR-grade water. Sequencing was performed on a 454 GS-FLX Titanium sequencer (454 Life Sciences) at the Penn State University Genomics Core Facility.

Sequences were processed using Mothur v1.44 [29] generally following the analysis example provided on the Mothur website (www.mothur.org, accessed on 14 December 2020). Sequences were trimmed of primers and barcodes and processed for quality with an average quality score of 35 for a window of 50 bp. Sequences that did not meet these quality requirements were discarded. Sequences were dereplicated and aligned to the v1.38 Silva non-redundant database [30] of bacterial and archaeal sequences. Sequences were screened to remove those that did not align and were clustered using a single-linkage algorithm allowing, at most, 2 differences between sequences in a cluster and the dominant sequence of that cluster. These clusters were used to check for chimeras using the Uchime algorithm [31] and presumptive chimeric sequences were removed. Sequences were classified with a naïve Bayesian classifier [32] using the Silva database (v1.38), and sequences classified to Eukaryota, Mitochondria, Chloroplast, or “Unknown” were removed. A pairwise distance matrix was calculated, and sequences were clustered into operational taxonomic units (OTUs) at a 97% similarity using the OptiClust algorithm [33]. A matrix representing the number of sequences in each OTU present in each sample was created by subsampling 3761 sequences from each community, which represented the minimum number of sequences present in any sample.

The alpha and beta diversity of the microbial communities in the samples was assessed to determine community characteristics and the extent of community similarity. There was a total of three samples (Top, Bottom, and Effluent) from each of the small reactors (AD 1–4, Bog 1–2, Ctl 1–2), along with samples from the original Bog and AD communities used to inoculate the small reactors. All samples were rarefied without replacement to determine the extent of sampling, and estimates were obtained for Good’s Coverage along with Chao1, Abundance Coverage Estimator (ACE), Shannon–Wiener, and Inverse Simpson indices to assess alpha diversity for each community. The shared distance between samples was measured using the Jaccard and Bray–Curtis calculators. The Jaccard calculator is based on the presence/absence of OTUs in each sample and is a measure of differences in community membership. The Bray–Curtis calculator is based on the abundance of each OTU per sample and is a measure of differences in community structure. The Jaccard calculator emphasizes the contributions of rare OTUs, whereas the Bray–Curtis calculator diminishes the contributions of rare OTUs. These distance measurements were used to test for significant differences between communities using AMOVA [34,35] and HOMOVA [36] with 10,000 iterations. Neighbor Joining was used to cluster samples, and resulting trees were visualized with Mega X [37]. Phylogenetic trees of representative OTU sequences in each sample were calculated using the Neighbor Joining algorithm as implemented in MegaX [37] and used for hypothesis testing with unweighted UniFrac [38].

## 3. Results

### 3.1. Chemical Analysis

The chemistry of the small reactors was assessed by measuring the pH, COD removal, daily gas production and composition, and VFAs in the effluent (Table 1). The effluent pH and evolved biogas of the small reactors were followed for approximately 4.5 months prior to the biomass sampling for DNA sequencing (Figure S1). During this period, the pH and evolved biogas of the small reactors inoculated from the large anaerobic digester (AD 1–4) decreased, whereas the pH and biogas production of the Bog-inoculated (Bog 1–2) reactors increased. The lag period for Bog 1–2 reactors to begin producing methane from the consumed COD was quite long (>250 d), and Bog 1 demonstrated greater COD removal and gas production in the period leading up to biomass sampling (Figure S1). Ctl 1–2 did not show evidence of microbial activity for the first 100 d of operation, but when fermentation began, the duplicate reactors demonstrated divergent chemical characteristics from each other. Ctl 2 maintained a lower pH, indicative of a primarily fermentative community, whereas Ctl 1 had a higher pH and produced some methane. The Bog and Ctl reactors were not inoculated from a pre-adapted community, and therefore, it would have taken longer for the microbial community to become established. The Bog 2 reactor especially did not appear to have reached steady-state conditions when biomass sampling occurred (Figure S1). In contrast, AD 1–4 demonstrated greater pH, COD removal rate, and methane production, as well as more stable operation, than the other reactors. In addition, these replicate reactors were more similar in performance to each other than the replicate Bog reactors were. A carbon balance for the small reactors showed that most of the carbon released from the digested substrate was present as inorganic dissolved carbon (Table S2).

Paired *t*-tests were performed for the reactor chemical parameters, including effluent COD, pH, daily gas volume (mL/d), methane concentration in the biogas (%), and total effluent VFAs (mM) (Table S3). Significant differences among the inoculated reactors were noted for COD removal rates, pH, and effluent VFAs, but not for daily gas volume or methane concentrations. For COD removal rates, the AD 1–4 reactors were significantly different with all the other reactors with the following two exceptions: AD 3 compared to Bog 1 and AD 1 compared to AD 4. This is consistent with the performance of the AD 1 and AD 4 reactors, which showed the highest average pH and lowest total VFAs, suggesting a more complete conversion of organic matter to biogas. Additionally, AD 3 was the poorest performing AD reactor and Bog 1 was the best performing Bog reactor; therefore, their similarity in COD removal rates is consistent with other chemical data, including pH and methane production. For pH, Bog 2 and AD 3 were different to AD 1, AD 2, and AD 4, but not with each other. The effluent VFAs were significantly different for AD 4 when compared with AD 1–3 or Bog 1–2, due to the very low VFAs for this reactor. Additionally, Bog 1–2 had higher VFAs and were, therefore, significantly different when compared with the AD 1–3 reactors, except for the comparison of Bog 1 with AD 3. In general, Bog 1 and AD 3 have similar chemical profiles, as do AD 1 and AD 4. AD 2 is somewhat in between these two pairs, and Bog 2 is very different to the other inoculated reactors.

### 3.2. Bioinformatic Analysis

**Figure 2:**
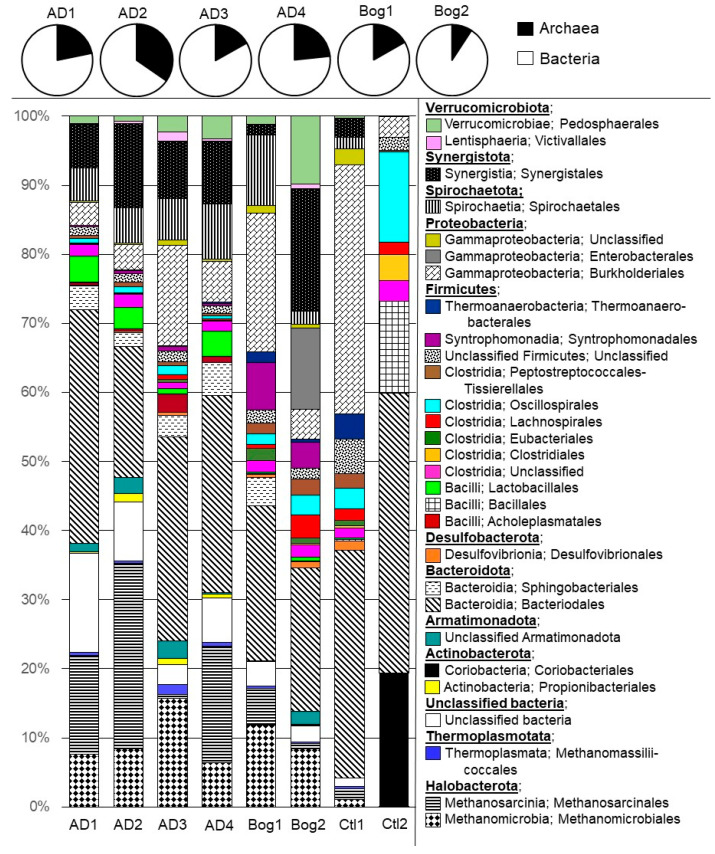
Microbial community of each small reactor representing OTUs that compose at least 1% of the reactor community. The V4 region of the 16S rRNA gene was sequenced using GS FLX (454 Life Sciences, Inc., Branford, CT, USA) technology. Sequences were processed using Mothur 1.44.3 [29] and classified with a naïve Bayesian classifier [32] using v1.38 of the non-redundant Silva database [30]. Four reactors (AD 1–4) were inoculated from a laboratory fixed-film, plug-flow anaerobic digester, two reactors were inoculated from an acidic peat bog (Bog 1–2) or were uninoculated (Ctl 1–2).

The small reactors had been operated for 307 days when biomass was sampled for DNA extraction and sequencing. Microbial communities were sequenced from the following three locations in the small reactors: biofilm from plastic media located at the top of the reactor near the effluent (“Top”), biofilm from plastic media located at the bottom of the reactor near the influent (“Bottom”), and liquid effluent collected from the reactor (“Effluent”). Alpha diversity showed a Good’s Coverage ranging from 96.6 to 99.3% for all 24 reactor samples along with the Bog and large AD inocula (Table 2). The rarefaction curves suggest the majority of community diversity was captured via sequencing for the small reactors, but not for the two inocula (Figure S2). The rarefaction curves echo the calculated alpha diversity indices that show higher diversity for the two inocula than for the small reactor communities. The structure of the small reactor microbial communities is shown in Figure 2. Despite efforts to maintain sterile reactor conditions, control reactors developed fermentative communities that were quite different from each other and from the inoculated reactors. Ctl 2 never produced methane and no archaeal sequences were obtained from this reactor. The community of Ctl 2 was dominated by sequences from *Bacteriodales*, *Coriobacteriales*, and *Bacillales* (40.5, 19.2 and 13.3% of sequences, respectively). Ctl 1 had sequences representing microbial orders seen in the inoculated small reactors, including some methanogens, but was generally dominated by *Bacteriodales* (32.9%) and *Burkholderiales* (36.1%) and produced almost no methane (Table 1). The development of microbial communities in uninoculated reactors underlines the difficulties in maintaining operational sterility and suggests automated processes should be incorporated into life support systems whenever possible. Although the control reactors were contaminated, no evidence of microbial activity was observed for over 100 days, suggesting the initial setup and operation of the reactors was performed under sterile conditions, which would have allowed for the establishment of microbial communities in the inoculated reactors. Additionally, the biomass of the inoculum for the AD and Bog reactors would be significantly larger than any incidental contamination; therefore, any influence of contamination on the microbial communities resulting in these reactors is likely to be small.

For the inoculated small reactors, Archaea represented 9–35% of the sequences, and were mainly composed of *Methanoculleus* within *Methanomicrobiales* (6.3–14.8%), and *Methanosarcina* within *Methanosarcinales* (0.8–26.1%), with fewer members of *Methanomassillicoccales*. Archaea in reactors AD 1–4 were dominated by acetoclastic *Methanosarcina,* whereas Archaea in Bog 1–2 were dominated by hydrogenotrophic *Methanoculleus*. The large number of sequences assigned to *Archaea* could be due to the choice of primers, which were designed to broadly amplify both *Bacteria* and *Archaea* [28]. The results from amplicon sequencing with these primers paralleled the metagenomic sequencing of the same samples, suggesting their accuracy in amplifying a broad range of sequences [28]. Similarly, high numbers of archaeal sequences were obtained by Han et al., with 38% of the sequences in one reactor inoculum assigned to the *Archaea* [16], and by Peces et al., who found 34% of all the sequences from digesters assigned to *Archaea* [18]. The sequences that could not be identified beyond the domain of bacteria represented 2.3–14.8% of the sequences, but were in larger numbers in reactors AD 1, 2, and 4 (than Bog 1–2 (2.3–3.4%).

The major phyla detected in inoculated small reactors included *Halobacterota* (12.5–39.6%) and *Thermoplasmatota* (0.3–1.5%) within the Archaea, and *Bacteroidota* (23.7–39.5%), *Firmicutes* (11.2–26%), *Synergistota* (1.5–24.2%), *Proteobacteria* (3.8–22.8%), *Verrucomicrobiota* (1.2–14.4%), *Spirochaetota* (2.7–11.5%), *Armatimonadota* (0.1–2.7%), *Actinobacteriota* (0.1–2%), and *Desulfobacterota* (0.2–1.3%) within the *Bacteria* (Figure 2). Most bacterial sequences were assigned to the classes *Clostridia*, *Bacilli*, and *Bacteroidia*, which are common, and often abundant, members of anaerobic digesters [13,14,15]. Members of these classes are metabolically diverse and participate in the hydrolysis and fermentation steps of anaerobic digestion, degrading all the major macromolecules [39,40]. The phyla *Chloroflexi* and *Proteobacteria* are also common fermentative members of the anaerobic digester microbiome, but their abundance is not as consistently dominant as the aforementioned phyla *Bacteriodetes* (*Bacteroidia*) and *Firmicutes* (*Clostridia* and *Bacilli*). The *Anaerolineaceae* family, the dominant *Chloroflexi* in this study, are active fermenters with a filamentous morphology that leads to their abundance in granular or fixed-film reactors [41]. The *Proteobacteria* were nearly entirely represented by members of *Gammaproteobacteria*. The orders *Burkholderiales* and *Enterobacterales* within *Gammaproteobacteria* represent metabolically flexibility capable of the hydrolysis and fermentation of a wide range of macromolecules [42,43]. Within *Verrucomicrobiota*, order *Oligosphaerales* within class *Lentisphaerae*, and *Pedosphaerales* within the class *Verrucomicrobiae* are polysaccharide fermenters and contribute to the breakdown of lignocellulosic material [44]. The *Spirochaetota* phylum was composed almost entirely of members of order *Spirochaetales* within the class *Spirochaetia*. *Spirochaetales* are carbohydrate fermenters that are especially abundant in reactors treating lignocellulosic material [45]. Phylum *Synergistota* was dominated by sequences attributed to the order *Synergistales* within the class *Synergistia*. *Synergistales* was entirely composed of members of the *Synergistaceae* family, a clade of fatty acid oxidizers that form syntrophic relationships with hydrogenotrophic methanogens as well as acetotrophic *Methanosaeta* [46]. Archaea were nearly entirely methanogens, with the order *Methanomicrobiales* mostly composed of hydrogenotrophic *Methanoculleus* and the order *Methanosarcinales* composed of *Methanosarcina* and acetotrophic *Methanosaeta*. *Methanosarcina* are the most metabolically versatile methanogens, able to use all three major methanogenic pathways [12].

Small reactors inoculated from the large AD reactor showed some changes in the microbial community structure when compared with the community of the large reactor. For instance, AD 1–4 were enriched in Archaea with 16.8–34.6% of sequences assigned to this domain as compared to 10.7% for the inoculum. In both the inoculum and AD 1–4 reactors, Archaea were dominated by the *Halobacterota*, although this number was lower in AD 3 than for AD 1, AD 2, and AD 4. Additionally, 54.6% of the archaeal sequences of the AD inoculum belonged to filamentous *Methanosaeta* compared to only 1–2% in AD 1–4. Within the Bacteria domain, AD 1–4 were enriched in *Bacteroidota* (31.3–46.6%) and *Proteobacteria* (4.5–17.4%) compared to the inoculum (26% and 0.7%, respectively). AD 1–4 had fewer bacterial sequences assigned to the Firmicutes (12.9–15.9%) and to the *Spirochaetota* (6–7.6%) compared to the inoculum (33.2 and 11.5%, respectively). Additionally, in the AD inoculum, 2.6% of bacterial sequences were assigned to the *Thermotogota*, but this phylum was not detected in AD 1–4.

In Bog 1–2, the changes in the microbial community from the inoculum to the small reactors were much more dramatic than for the AD 1–4 reactors. The Bog inoculum was exceptionally diverse with sequences assigned to 48 different phyla compared to sequences assigned to 18 phyla in Bog 1 and 24 phyla in Bog 2. The microbial communities of Bog 1–2 were much lower in community diversity and evenness than the inoculum (Table 2) and were more similar to AD 1–4 for these characteristics. For the bog inoculum, 9.1% of sequences were assigned to the *Archaea*. Of these, 73.6% were *Crenarchaeota*, 26% were *Bathyarchaeota*, 3.5% were *Halobacterota*, and 0.2% were *Euryarchaeota*. Although the Bog 1–2 communities had a similar number of archaeal sequences, over 98% were *Halobacterota*. As methanogens are found in *Halobacterota* and *Euryarchaeota*, inoculation of the small reactors resulted in a dramatic enrichment for methanogens in the small reactors. There were few dominant bacterial phyla in the Bog inoculum, but 35.6% of bacterial sequences were assigned to *Acidobacteriota*. However, only two sequences assigned to this phylum were detected in Bog 1–2. Additionally, sequences assigned to the *Chloroflexi* (2.8%) and *Desulfobacterota* (3.2%) in the Bog inoculum decreased in Bog 1–2 (<0.05% and 0.4–1%, respectively). The small reactors were enriched in *Bacteroidota* (22.5–30.6%), *Firmicutes* (20.6–23.3%), and *Spirochaetota* (2.1–13.5%) compared to the inoculum (4.3, 0.4, and 0.6%, respectively). *Synergistota* was 1.7–19.2% of the Bog 1–2 bacterial community, despite not being detected at all in the Bog inoculum.

**Figure 3:**
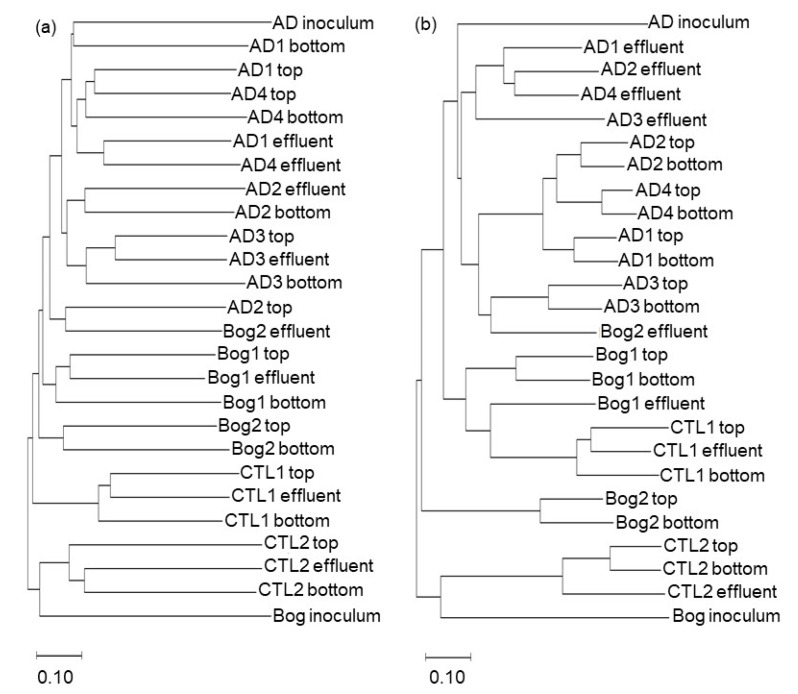
Clustering of distances of microbial communities sampled from three locations (Top, Bottom, Effluent) in small reactors. Four small reactors were inoculated from a laboratory fixed-film, plug-flow anaerobic digester (AD 1–4), two from an acidic peat bog (Bog 1–2) or were uninoculated (Ctl 1–2). DNA was extracted from three locations for each reactor (Top, Bottom, and Effluent), and from the inoculum for the Bog and AD reactors, and 16S sequences were clustered into OTUs with a shared similarity of 97% using the Mothur 1.44.3 program [29]. Distances between communities were calculated using a Jaccard (**a**) or Bray–Curtis (**b**) algorithm and clustered using the Neighbor Joining method in MegaX [37]. Trees were visualized with MegaX.

The pairwise distances between the sample communities were calculated using the Jaccard and Bray–Curtis measurements and used for ordination and clustering. The data were highly dimensional, and neither Principal Coordinates Analysis (PCoA) nor Non-Metric Dimensional Scaling (NMDS) was able to resolve a significant portion of the variance in only two axes. For instance, PCoA with the Jaccard distance matrix assigned 14.9% of variance to the first axis with an additional 10.4% of variance described by the second axis for a total of 25.3%. PCoA with the Bray–Curtis distance matrix assigned 28.2% of variance to the first axis and 16.4% to the second axis for a total of 44.7%. NMDS with two dimensions using the Jaccard matrix had a stress of 0.35 with an R^2^ of only 47.7; adding a third dimension dropped the stress to 0.25 with an R^2^ of 49.0. Similarly, the lowest stress found for NMDS with two dimensions using the Bray–Curtis matrix was 0.26 with an R^2^ of 71.0%, and with three dimensions, the lowest stress was 0.17 with an R^2^ of 81.0%. Ideally, to apply ordination to resolve differences in communities, the vast majority of the variance among communities should be described in two dimensions (PCoA), and stress should be below 0.20 (NMDS). Therefore, we decided against ordination to show the relationships among communities and used clustering instead. Communities were clustered using the Jaccard and Bray–Curtis distance matrices using the Neighbor Joining algorithm (Figure 3). Clustering based on Jaccard distances generally clustered samples from the same reactor together. Bog-inoculated (Bog 1–2) and Control (1–2) reactor samples clustered with the Bog inoculum, and anaerobic digester-inoculated reactor (AD 1–4) samples clustered with the large reactor inoculum (AD inoculum). The clustering of the small reactor samples was similar based on the Bray–Curtis distances. The NMDS ordination of small reactor communities based on the Bray–Curtis distance matrix is shown in Figure S3. Genera with *p*-values below 0.0001 are overlain to show their influence on the ordination. Despite the high stress value of this ordination, the results mimic the clustering with the Ctl 1, Ctl 2, and Bog inoculum samples further removed from the rest of the samples, which are located close together.

The distance matrices created with either Jaccard or Bray–Curtis metrics were also used with AMOVA, HOMOVA, and unweighted UniFrac to test for statistically significant differences in the community structure and membership between the pairs of samples. Pairwise comparisons of samples were used to determine the significance of the following two variables that could affect community composition: the inoculum source (AD, Bog, or Ctl), and location in the small reactor (Top, Bottom, Effluent). A total of 26 samples were used for hypothesis testing, which included three samples (Top, Bottom, and Effluent) from each of the eight small reactors, along with the two inocula. For the inoculated reactors (AD 1–4 and Bog 1–2), statistically significant differences (*p* < 0.05) were only detected using AMOVA with the Bray–Curtis distances for Effluent samples compared to Top or Bottom samples. This finding suggests there may have been subtle differences in community structure between the Effluent and Top/Bottom samples. However, AMOVA tests using Jaccard distances, which increases the weight of rare species, were not significant; therefore, it is unlikely that there were substantial differences in the community membership based upon sample location. This echoes the results of the cluster analysis that showed samples clustered based upon the reactor, and not the location, from which they were sampled (Figure 3). Comparisons were also made for samples from the small reactors originating from different inocula (i.e., AD, Bog, Ctl). AMOVA with either Bray–Curtis or Jaccard distances showed significant differences in communities based upon the inoculum source (i.e., AD vs. Bog, AD vs. Ctl, Bog vs. Ctl reactors). Unweighted Unifrac showed significant differences between the AD and Bog, and between the AD and Ctl reactor communities, but not between the Bog and Ctl reactor communities. This suggests the presence of community members unique to the AD reactors, and also suggests that the contamination of the Ctl reactors originated from the Bog reactors. AMOVA and UniFrac tests suggest that sample communities were significantly different based upon the inoculum source. The HOMOVA tests did not detect any differences.

Lastly, the reactor communities of the eight small reactors were tested to determine the reproducibility of microbial community formation in replicate reactors inoculated from the same source. Samples were pooled by reactor yielding a total of eight samples (AD 1–4, Bog 1–2, Ctl 1–2). Rarefaction curves were calculated for the merged samples (Figure S2). The small reactors and the AD inoculum show a leveling off of novel sequences. However, the Bog inoculum curve continues to climb, suggesting not all the diversity from the sampling has been captured, despite a Good’s coverage of 97.3% (Table 2). This is in line with the extremely high alpha diversity calculated for this inoculum (Table 2). HOMOVA with either Jaccard or Bray–Curtis distances detected differences between the two inocula, and between either of the two inocula and any of the small reactor communities. HOMOVA is sensitive to detecting when one community is a subset of another [47]. This suggests the community of the AD inoculum, which was taken from the large reactor, may be a subset of the Bog inoculum. Furthermore, the resultant communities inoculated from each source were also a subset of the initial inoculum. However, there were no significant differences for any pairwise comparisons between any small reactor communities using HOMOVA. AMOVA detected a few differences between the small reactor communities, but not between the two inocula. With Jaccard distances, AMOVA detected differences between AD 1 and either the AD or Bog inoculum, between AD 1 and AD 3, and between AD 1 and Ctl 1. With Bray–Curtis distances, AMOVA only detected significant differences between AD 4 and Ctl 2. For unweighted UniFrac with Jaccard differences, the results nearly mirrored those obtained with HOMOVA in that each inoculum was significantly different than any of the small reactor communities. However, the two inocula were not significantly different from each other. The results for the unweighted UniFrac with Bray–Curtis distances were nearly the same as those for Jaccard distances, with some exceptions. The AD inoculum community was significantly different than the communities for AD 1, 3, and 4, for Bog 1, and for Ctl 1–2, whereas the Bog inoculum community was significantly different when compared with any of the other small reactor communities.

The core members of the microbiome among all the reactors were assessed using a cutoff of a minimum of 1% abundance of the community. With this parameter, there were no shared OTUs among all eight reactors. This is not particularly surprising given that OTUs, on average, have no more than 3% divergence among all the members; therefore, several OTUs may represent a single species. Additionally, it is likely that several OTUs were represented among all the reactors, but that abundance was less than the 1% cutoff. When the two control reactors were removed from the analysis, there were four OTUs shared among the remaining six reactors, AD 1–4 and Bog 1–2. These four OTUs included one that could not be classified past the order *Bacteroidales*, one that could not be classified past the order *Burkholderiales*, one from the *Sphaerochaeta* genus, and one from the *Methanoculleus* genus. One OTU was shared only among the AD 1–4 reactors and was classified to the DEV114 genus within *Pedosphaeracea*. Five OTUs were shared only between the Bog 1–2 reactors. Of these OTUs, one could not be classified past the *Firmicutes* phylum, one could not be classified past the *Rikenellaceae* family, one could not be classified past the *Methanomicrobiaceae* family, one was a member of the *Microvirgula* genus, and one was a member of the *Sphaerochaeta* genus.

**Figure 4:**
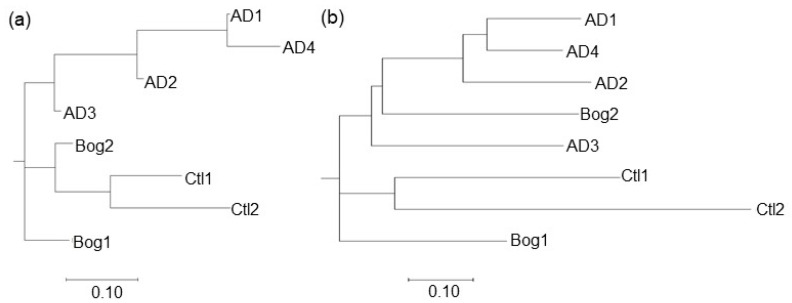
Clustering of distances for small reactors based on chemical data (**a**) and microbial communities; (**b**). Four small reactors were inoculated from a laboratory fixed-film, plug-flow anaerobic digester (AD 1–4), two from an acidic peat bog (Bog 1–2) or were uninoculated (Ctl 1–2). DNA was extracted from three locations for each reactor, and 16S sequences were clustered into OTUs with a shared similarity of 97%. Sequences from the three sampling locations were merged for each reactor. Chemical data from 12 parameters (pH, gas produced, %CH4, %H2, %CO2, effluent COD, and VFA concentrations for acetate, propionate, isobutyrate, butyrate, isovalerate, and valerate) were normalized to the maximum value for that parameter from the eight reactors. Distances between communities for both microbial community and chemical data were calculated using the Bray–Curtis algorithm and clustered using the Neighbor Joining method in MegaX [37]. Trees were visualized with MegaX.

Additionally, the differences between the reactors were calculated based on the chemical data. There was a total of twelve chemical parameters used as variables for each site: pH, %COD removal rate, total daily gas production in mL, % methane, % carbon dioxide, and % hydrogen in the headspace, and the concentrations in mM of acetate, propionate, isobutryate, butyrate, isolvalerate, and valerate in the effluent. Each variable was normalized to the maximum value of that variable in any reactor, yielding a variable response between 0 and 1. In this way, each variable provided the same weight to the clustering algorithm. The microbial communities for each reactor represented the merging of community data from the three sampling locations (Top, Bottom, and Effluent). The shared distances between the reactors for both the chemical data and the merged microbial communities were calculated using the Bray–Curtis algorithm and clustered using Neighbor Joining (Figure 4). The Jaccard algorithm was not used for this analysis as it is a measure of shared OTUs between communities, and appropriate distance measures could not be calculated with only 12 variables. The clustering of reactors based on either the microbial community or the chemical data were similar. Reactors AD 1, 2, and 4 clustered together for both community and chemical data, and Bog 1–2 clustered together for both data types. Reactor Ctl 2 showed the greatest distance for both data types. The Bog reactors clustered with AD 3 and Ctl 2 with chemical data due to the low methane content of the biogas, low COD removal rates, lower pH, and higher VFAs.

## 4. Discussion

This work studied the influence of the inoculum and operational conditions on the establishment of microbial communities in plug-flow, fixed-film reactors. In the current study, a marked decrease in diversity was noted in comparing the lab-scale reactors to the inocula, and this was most dramatic for the Bog-inoculated reactors. Previous studies have demonstrated a similar decrease in community diversity when comparing lab- to full-scale reactors [16,17]. Scaling down the anaerobic digestion process contributes to a loss of microbial community complexity as full-scale reactors are more likely to harbor environmental niches that can support greater microbial diversity. Loss of diversity may lead to a loss in the ability of the microbial community to respond to changes in the reactor, for example, from periodic overloading or a drop of temperature due to a loss of reactor heating. However, high microbial diversity does not necessarily impart an increase in substrate processing, which is the most important function for a digester in a life support system. Venkiteshwaran et al. seeded triplicate reactors from each of 50 full-scale reactors and sequenced the resulting 150 microbial communities [48]. The researchers found no correlation between the methane production rates and the community indices of diversity or evenness [48]. Therefore, a highly diverse microbial community does not necessarily predict efficient reactor performance.

In general, the diversity of the lab-scale reactor community may result from functional redundancy provided by phylogenetically different microbes. Most studies examining the development of communities in lab-scale reactors inoculated from different sources have found a convergence in both the functioning and community structure [16,17,18,49], suggesting deterministic processes primarily drive microbial community development. A study by Peces et al. found that lab-scale digester communities demonstrated different metabolic rates for stages of anaerobic digestion (e.g., hydrolysis, acetogenesis, methanogenesis) during the start-up period, but that these rates converged over time for reactors inoculated from different sources [18]. Microbial community convergence also occurred, yet the differences in community structure and membership based on the inoculum source appeared to be due to minor community members [18]. Lemoine et al. also saw persistent differences in the community structure based on the inoculum source despite a convergence in the methane production potential for these reactors [17].

Lemoine et al. suggested that the use of a pre-adapted inoculum, coupled to a slow start-up period, led to the high reproducibility of performance and resultant communities in the replicate reactors in their study [17]. Indeed, the use of a high diversity or non-adapted inoculum may allow stochastic processes to cause divergences in microbial communities and performance in replicate reactors. Vanwonterghem et al. inoculated triplicate reactors from a mixture of lake sediment, rumen fluid, and anaerobic lagoon material and followed performance and microbial community structure over time. The researchers detected different members of the microbial communities in replicate reactors as responsible for similar processes (e.g., hydrolysis, fermentation, acetogenesis, methanogenesis), leading to different community structures and somewhat different reactor performances [50]. Other studies examined the development of methanogenesis in lab-scale reactors inoculated from wholly environmental sources. Braun et al. used PAH-contaminated soil, sediment, or wastewater treatment plant sludge as the inoculum for a single lab-scale reactor [51]. The three reactors possessed different microbial communities and demonstrated vastly different performances, with the reactor receiving an adapted inoculum (wastewater treatment plant sludge) demonstrating high methane production potential and PAH removal [51]. Perrotta et al. inoculated anaerobic digesters with camel manure, mangrove intertidal sediment, or sludge from a full-scale anaerobic digester [52]. Despite an enrichment of common OTUs among lab-scale reactors, suggesting some convergence of communities, the reactors retained structurally different communities and demonstrated different performance [52].

Although it is evident that an environmental inoculum would not be adapted to a constructed reactor environment, thus leading to stochasticity in microbial community formation, it is also important to match the reactor inoculum to the substrate. For example, Moestedt et al. inoculated duplicated reactors from two full-scale anaerobic reactors treating either food and slaughterhouse waste or digesting municipal sludge [49]. One reactor from each inoculum was then fed food waste as a substrate while the other reactor in the pair was fed digested sludge. The reactors where the inoculum and substrate were matched showed better initial performance, although the performance and reactor communities became similar [49]. Therefore, the reproducibility and predictability of performance in an anaerobic digester would best be obtained from an inoculum obtained from similar reactor type and operating conditions. In the current study, the replicate Bog reactors had very different community structures and performances from each other than did the replicate AD reactors. The large lab-scale anaerobic reactor was operated for 455 days before serving as an inoculum source for AD 1–4, and therefore, had already selected as a subset of the initial inoculum that could thrive in a lab-scale reactor environment. However, AD 3 had a different community and poorer performance than the other AD reactors. Although there were detectable differences in the microbial communities in the four reactors, these community shifts were smaller and the microbial community development more reproducible than in the Bog-inoculated reactors due to the use of an inoculum that had already been adapted to a reactor environment.

It is notable that the reactors inoculated from a highly diverse, acidic bog community could eventually develop communities capable of methanogenesis from ersatz waste, which is quite different from the lignocellulosic material that is the primary substrate in the bog environment [27]. Although the communities in Bog 1 and Bog 2 differed from each other, they were more similar to AD 1–4 than the original inoculum. Additionally, the methane production from Bog 1 was similar to that of AD 3, suggesting that a community capable of efficiently processing the ersatz waste could be obtained from a wholly environmental inoculum. These results provide a measure of assurance that an anaerobic digester in a life support system could be restarted, in the event of reactor failure, from a preserved inoculum or even a naturally occurring source (e.g., human waste).

In this study, deterministic processes, such as operational conditions and choice of substrate, led to a convergence among the small reactor communities inoculated from different sources. However, stochastic processes resulted in somewhat different communities in replicate reactors inoculated from the same source. In this study, clustering analysis of triplicate samples from each small reactor generally grouped according to the reactor from which they were taken as well as by the inoculum source. Despite the clustering analysis, AMOVA detected few statistical differences between the communities in the small reactors, and HOMOVA detected no differences at all. Additionally, the unweighted UniFrac suggested some differences between the small reactor communities, but differences were not detected with the weighted UniFrac. These results suggest minor community members were responsible for the differences in community structure by inoculum source. It is not known if these differences would eventually disappear over longer operational periods as there is generally some stochasticity in community structure during initial reactor operating periods [16,18]. As previously discussed, reactors with somewhat different methanogenic communities can demonstrate a similar performance due to functional redundancy, and different sequencing efforts have resulted in different “core” microbiomes of anaerobic digesters [13,14,15]. Given the resolution of the current sequencing methods, it is possible that some of the differences detected in methanogenic communities are insignificant in terms of actual reactor performance, whether due to functional redundancy, normal temporal fluctuations in community membership, or the limitations of reactor sampling for sequencing.

Life support systems that utilize microbial communities to carry out specific functions require predictability, reproducibility, and redundancy. The anaerobic rather than aerobic treatment of organic waste is advantageous as the produced methane and CO_2_ can be further utilized for energy or food production without oxygen consumption. Extensive research has been devoted to the European Space Agency’s MELiSSA project [5,6], which depends upon microbial systems to accomplish waste treatment and food production, thereby demonstrating how microbial reactor predictability can be accomplished through control of the reactor community and operational conditions. The translation of this work to a methanogenic community may require the formation of an artificially constructed, pathogen-free inoculum composed of community members providing all the necessary metabolic flexibility to process a complex waste stream. Reducing the number of community members would reduce the stochastic nature of microbial community formation, although reducing the diversity could make the reactor more sensitive to process upsets. However, this could be countered by operating multiple reactors in parallel, and maintaining preserved inoculum for restarting reactors, if necessary. Additionally, reactor operation could implement heat pretreatment to sterilize the influent waste that would also hydrolyze lignocellulosic material, thus making it easier to mineralize in the reactor [53]. Methanogenic reactors would thus serve a vital function in life support systems by facilitating the mineralization and recycling of carbon and nutrients.

## Figures and Tables

**Table 1:** Effluent chemical characteristics of small anaerobic reactors. Reactors AD 1 through AD 4 were inoculated with the large anaerobic digester effluent, reactors Bog 1 and 2 were inoculated from acidic bog sediment, and control reactors (Ctl 1 and 2) were uninoculated. %COD refers to the % removal of COD in the reactor. Gas concentrations are noted unless they were below the limit of quantitation (LQ) for the method. VFAs refer to the sum of acetate, propionate, butyrate, isobutyrate, isovalerate, and valerate effluent concentrations.

	AD 1	AD 2	AD 3	AD 4	Bog 1	Bog 2	Ctl 1	Ctl 2
pH	7.8	7.7	7.5	7.9	7.6	7.2	7.2	6.0
%COD	87.1 ± 0.8	71.3 ± 2.3	57.1 ± 3.6	84.3 ± 3.6	50.3 ± 2.8	29.3 ± 1.7	32.1 ± 2.8	26.8 ± 2.1
Gas (mL/d)	27.0 ± 5.28	30.1 ± 6.6	30.7 ± 5.0	29.8 ± 15.8	57.3 ± 10.5	27.8 ± 9.4	1.57 ± 2.0	2.81 ± 1.35
%H_2_	LQ	LQ	LQ	LQ	LQ	LQ	LQ	24.4 ± 10.0
%N_2_	10.8 ± 0.5	11.0 ± 0.9	10.6 ± 0.9	10.2 ± 1.1	7.9 ± 0.8	12.1 ± 2.6	34.4 ± 2.7	28.8 ± 3.6
%CH_4_	25.3 ± 1.4	25.9 ± 4.5	23.3 ± 4.7	26.2 ± 3.3	21.2 ± 4.7	21.2 ± 7.2	3.3 ± 2.4	LQ
%CO_2_	63.9 ± 7.7	63.1 ± 7.6	66.1 ± 7.8	63.6 ± 6.6	70.9 ± 3.6	66.7 ± 3.6	62.3 ± 17.2	46.8 ± 8.9
VFAs (mM)	25.0 ± 4.6	57.7 ± 3.3	80.6 ± 6.1	5.1 ± 2.1	100.4 ± 6.1	118.8 ± 9.2	126.7 ± 5.7	68.6 ± 3.8

**Table 2:** Diversity indices calculated for small reactor communities. Sequences were clustered into OTUs at 97% similarity, and alpha diversity was calculated using the “summary.single” command in the Mothur 1.44.3 program [29]. Sobs = observed number of OTUs, Good’s = Good’s coverage estimator, Shannon = Shannon–Wiener diversity index, Inv. Simpson = Inverse Simpson index, Chao1 = Chao1 index, ACE = Abundance Coverage Estimator. One standard deviation is shown for Shannon indices, and the upper and lower 95% confidence intervals are shown in parentheses for Inverse Simpson, Chao1, and ACE indices.

Sample	Sobs	Good’s	Shannon	Inv. Simpson	Chao1	ACE
AD inoculum	325	97.4%	4.26 ± 0.05	33.62 ± 1.95	562 (476, 697)	694 (621, 786)
Bog inoculum	790	97.3%	5.32 ± 0.06	68.57 ± 6.06	1840 (1596, 3072)	3072 (2829, 3343)
AD 1-top	194	97.3%	2.96 ± 0.06	8.36 ± 0.44	442 (332, 637)	558 (476, 663)
AD 1-bottom	201	97.4%	3.09 ± 0.06	8.33 ± 0.53	353 (289, 464)	522 (451, 615)
AD 1-effluent	224	97.3%	3.20 ± 0.06	7.59 ± 0.55	434 (345, 589)	505 (439, 592)
AD 2-top	209	97.2%	3.21 ± 0.06	9.03 ± 0.65	452 (348, 637)	622 (537, 729)
AD 2-bottom	184	98.0%	2.93 ± 0.06	6.82 ± 0.44	261 (227, 323)	357 (311, 422)
AD 2-effluent	252	96.6%	3.53 ± 0.05	16.35 ± 0.80	463 (382, 593)	628 (548, 729)
AD 3-top	172	97.8%	3.11 ± 0.06	7.85 ± 0.59	307 (246, 417)	498 (422, 598)
AD 3-bottom	187	97.8%	3.32 ± 0.06	10.16 ± 0.71	304 (252, 398)	426 (366, 506)
AD 3-effluent	216	97.4%	3.62 ± 0.06	14.01 ± 1.11	432 (338, 599)	550 (477, 643)
AD 4-top	137	98.2%	2.45 ± 0.06	5.54 ± 0.26	289 (214, 437)	354 (295, 437)
AD 4-bottom	174	97.3%	2.27 ± 0.06	4.36 ± 0.19	404 (304, 579)	618 (520, 744)
AD 4-effluent	226	97.4%	3.51 ± 0.05	14.75 ± 0.92	388 (320, 504)	449 (393, 524)
Bog 1-top	189	97.9%	3.43 ± 0.05	14.51 ± 0.81	326 (263, 441)	385 (334, 454)
Bog 1-bottom	216	97.4%	3.73 ± 0.05	22.14 ± 1.18	396 (320, 528)	534 (463, 625)
Bog 1-effluent	188	97.9%	3.10 ± 0.06	7.78 ± 0.56	281 (240, 355)	368 (320, 435)
Bog 2-top	204	97.2%	3.52 ± 0.05	17.18 ± 0.97	427 (333,589)	685 (580, 820)
Bog 2-bottom	208	97.1%	3.35 ± 0.06	11.46 ± 0.73	476 (361, 675)	690 (590, 816)
Bog 2-effluent	201	97.6%	3.19 ± 0.06	7.94 ± 0.55	379 (300, 522)	455 (394, 535)
Ctl 1-top	159	98.1%	2.44 ± 0.07	3.47 ± 0.21	290 (229, 407)	375 (318, 453)
Ctl 1-bottom	150	98.5%	3.37 ± 0.05	14.66 ± 0.94	221 (185, 292)	280 (241, 337)
Ctl 1-effluent	135	98.8%	2.81 ± 0.06	6.22 ± 0.41	182 (157, 234)	232 (200, 278)
Ctl 2-top	126	98.3%	2.42 ± 0.06	5.36 ± 0.25	386 (244, 698)	380 (314, 470)
Ctl 2-bottom	103	98.7%	2.02 ± 0.06	3.35 ± 0.17	184 (141, 276)	239 (196, 301)
Ctl 2-effluent	65	99.3%	1.46 ± 0.05	2.79 ± 0.07	85 (73, 118)	124 (100, 165)

## Data Availability

The data presented in this study are openly available in the NCBI SRA.

## Supplementary Materials

The following are available online at https://www.mdpi.com/article/10.3390/life11121374/s1, Figure S1: Performance of small anaerobic reactors over a period of 140 days prior to sampling for DNA sequencing; Figure S2: Rarefaction curves for microbial communities from small reactors; Figure S3: NMDS ordination of the matrix of Bray-Curtis distances of small reactor microbial communities as calculated in Mothur v1.44.3; Table S1: Primers, barcodes, and accession numbers for sequencing data; Table S2: Carbon balance for small reactors showing consumed carbon and recovered carbon; Table S3: Paired *t*-test of chemical parameters for small anaerobic reactors.
