# Supplementary material for: Convergent Microbial Community Formation in Replicate Anaerobic Reactors Inoculated from Different Sources and Treating Ersatz Crew Waste
Source: Life (Basel). 2021 Dec 10;11(12):1374. doi: 10.3390/life11121374 (PMC8706314; doi:10.3390/life11121374)
Supplement: Supplementary file 1 [file life-11-01374-s001.zip › life-1396648-supplementary.pdf]

Supplemental Methods and Results

Supplementary methods

Small reactor construction and operation

Small, fixed-film, flow-through anaerobic reactors were constructed to test the reproducibility of biofilm formation in these reactors. These small scale reactors were constructed in a similar manner to the large reactor [1], using threaded cPVC tubing with dimensions of 12 ft (25.4 cm) in length and 1.5 in (3.8 cm) in diameter. A series of reduced bushings were threaded into the ends of the reactors and attached to a ¼-inch (0.64-centimeter) barbed tube fitting. Tubing was a size LS16 Tygon Masterflex food grade, B-44-4X formulation with low gas permeability (Saint-Gobain, Akron, OH). Reactors were operated in an upflow configuration with a peristaltic pump fitted with a multichannel pump head used to push fluid at 5 mL/min through the bottom of the reactor and out through the top. The effluent from the top of the reactor was dripped into a 60-milliliter plastic syringe barrel fitted with a #5 neoprene stopper with two ¼ in holes drilled into it. Tubing was passed through both holes in the stopper. One tube carried effluent from the reactor down into the syringe barrel. The other tube was placed near the top of the syringe barrel and used to passively collect evolved biogas into a Cali-5 bond gas sampling bag (Calibrated Instruments, Inc.). Holes in the stopper were sealed with silicone sealant to prevent gas leakage. The end of the syringe barrel was fitted with a 3-way Luer-Lok valve that was used for feeding the reactor. Approximately 10 mL of liquid was maintained in the 50-milliliter syringe barrel that allowed biogas to migrate out of the reactor effluent and into the syringe barrel where it could then enter the gas sampling bag. The reactors were fed three times per week. During feeding, the pump was turned off and the 10 mL of effluent in the syringe barrel was removed for chemical analysis through the 3-way Luer-Lok valve at the bottom of the syringe barrel. By turning the Luer-Lok valve, the flow pathway was changed from the bottom of the syringe barrel to an external port by which effluent could be removed and fresh medium could be added. A total of 10 mL of fresh, sterile medium was injected into the syringe barrel, and the 3-way valve was closed so that the fluid pathway was returned to one that exited the syringe barrel and entered tubing that went to the peristaltic pump and back into the bottom of the small reactor. Small reactors were 600 mL in volume, and received 60 mL of fresh medium per week with 60 mL of effluent wasted, resulting in an HRT of 70 d. Effluent pH was determined via a pH meter equipped with a Ag/AgCl probe. Evolved reactor gas was collected in Cali-5 Bond bags for the determination of gas volume and composition. Gas volume production was measured by drawing gas from the bags with an airtight graduated syringe. Gas composition was determined via gas chromatography with a model 8610C GC (SRI Instruments, Torrance, CA) equipped with a 6-foot (183-centimeter) MS13X molesieve column (Restek, Bellefonte, PA) and a helium ionization detector at a constant pressure of 23 psi. The temperature program included an initial temperature at 40° for 4.5 min, followed by a 40°C/min ramp to 220°C held at 3.5 min. Laboratory grade gases of methane, hydrogen, nitrogen, and carbon dioxide were used for external calibration. The COD of effluent from the reactors was determined with Chemetrics (Midland, VA) HR+ COD vials with a measurement range of 0–15,000 ppm using a COD standard (Chemetrics, Midland, VA). Volatile fatty acids (VFAs) were quantified on a Dionex LC30 equipped with an IonPac AS15 anion exchange column operated at 31°C. Anions were eluted with a gradient of potassium hydroxide from 2–25 mM over 20 min at a 1 mL/min flow rate with ECD and suppressor currents at 97 and 180 mA, respectively. A standard free volatile fatty acid mix (Sigma-Aldrich) was used for external calibration. Paired t-tests were performed to determine if the reactor chemistry was significantly different. The data used were collected from the six weeks prior to the collection of biomass for DNA extraction and sequencing. The data that were analyzed included pH, COD removal efficiency (%), daily gas volume produced (mL/d), methane composition of the evolved gas (%), and effluent VFAs (mM).

Bioinformatics and statistical analysis of reactors

Plastic media was removed from the small reactors on day 307 of the operation for biomass extraction and DNA sequencing. Approximately 1 g of plastic media was removed from the eight small reactors in the following three locations from each reactor: biofilm from plastic media located at the top of the reactor near the effluent ("Top"), biofilm from plastic media located at the bottom of the reactor near the influent ("Bottom"), and liquid effluent collected from the reactor ("Effluent"). Cells from the plastic media were collected by vortexing media in 25 mL of 0.1 M Tris buffer, pH 8.0, with 0.05 M NaCl, 0.2% Na-pyrophosphate, and 0.1% Triton-X 100, and collecting dislodged cells by centrifugation. Cells in effluent samples were collected by centrifugation without prior vortexing. Biomass DNA was extracted with a MoBio Power Soil DNA extraction kit (Mo Bio Laboratories, Carlsbad, CA). Triplicate DNA extractions were performed for each sample, and the extracted DNA from each port was pooled prior to analysis. DNA was extracted from biomass, and amplicon for 454 FLX sequencing was obtained using universal primers (Table S1)[2]. Sequences were processed using Mothur v1.44 [3] with sequences sharing a 97% similarity clustered into operational taxonomic units (OTUs). A matrix representing the number of sequences in each OTU present in each sample was created by subsampling 3,761 sequences from each community, which represented the minimum number of sequences present in any sample. The matrix of shared OTUs for the large reactors subsampled 9,188 sequences from each sample, which represented the minimum number of sequences for any of the six port samples.

Table S1. Primers, barcodes, and accession numbers for sequencing data. The V4 region of the 16S gene was sequenced using LS454 technology (Life Sciences). Data were submitted to the NCBI GenBank database under BioProject: PRJNA762280 and SRA: SRP336625.

Accession	Sample	Sampling	Barcode	Sample
SRX12142909	SAMN21383749	Large reactor, biomass extracted July 2010	TGATACGTCT	Port1
			TACTGAGCTA	Port3
			CATAGTAGTG	Port4
			CGAGAGATAC	Port6
SRX12142910	SAMN21383750	Large reactor, biomass extracted January 2011	ACGAGTGCGT	Port1
			AGACGCACTC	Port2
			AGCACTGTAG	Port3
			ATCAGACACG	Port4
			ATATCGCGAG	Port5
			CTCGCGTGTC	Port6
SRX12142911	SAMN21383751	Small reactors, biomass extracted November 2012	ACGAGTGCGT	Ctl1-top
			CTCGCGTGTC	Ctl1-bottom
			TAGTATCAGC	Ctl1-effluent
			ACGCTCGACA	Ctl2-top
			AGACGCACTC	Ctl2-bottom
			AGCACTGTAG	Ctl2-effluent
			ATCAGACACG	Bog1-top
			ATATCGCGAG	Bog1-bottom
			CGTGTCTCTA	Bog1-effluent
			TCTCTATGCG	AD2-top
			TGATACGTCT	AD2-bottom
			TACTGAGCTA	AD2-effluent
			CATAGTAGTG	Bog inoculum
			CGAGAGATAC	AD inoculum
SRX12142912	SAMN21383752	Small reactors, biomass extracted November 2012	ACGAGTGCGT	Bog2-top
			ACGCTCGACA	Bog2-bottom
			AGACGCACTC	Bog2-effluent
			AGCACTGTAG	AD1-top

	ATCAGACACG	AD1-bottom
	ATATCGCGAG	AD1-effluent
	CGTGTCTCTA	AD3-top
	CTCGCGTGTC	AD3-bottom
	TAGTATCAGC	AD3-effluent
	TCTCTATGCG	AD4-top
	TGATACGTCT	AD4-bottom
	TACTGAGCTA	AD4-effluent
Forward primer (V4 16S)	5′ AAACTYAAAKGAATTGRCGG 3′	
Reverse primer (V4 16S)	5′ ACGGGCGGTGTGTRC 3′	

Supplemental Results

Small reactors were fed weekly with a concomitant removal of effluent, and an assessment of pH, COD removal efficiency, and short-chain volatile fatty acids (VFAs). The volume and composition of evolved biogas was determined. The reactor performance was followed over a period of 140 days prior to collecting biomass for DNA extraction and sequencing (Figure. S1). Over the course of operation, AD1–4 reactors converged to a similar pH and gas production. Bog1–2 reactors increased in pH and gas production, but Bog 1 showed a greater pH and gas production than Bog2. Ctl1 and Ctl2 reactors became fermentative but produced little gas. Ctl1 had a much higher pH than Ctl2. A carbon balance was conducted for small reactors, consisting of total carbon fractions of soluble organic, dissolved inorganic, and gaseous phase carbon (Table S2). The total organic carbon was determined from Chemical Oxygen Demand (COD) measurements. The total gas phase carbon was determined from gas volume and composition measurements. The total dissolved inorganic carbon was determined from pH and gas composition measurements using a Henry's Law value of 0.034 mol/kg*bar [4] and freshwater constants for K_1 and K_2 of $10^{-6.3}$ and $10^{-10.3}$, respectively. Recovered carbon ranged from 59 to 181% of the carbon provided in feed. AD1–4 consumed more COD, converting it to gaseous phase CH_4 and CO_2, than the other reactors. For the small reactors, there were a total of three samples (Top, Bottom, and Effluent) from each of the reactors (AD1–4, Bog1–2, Ctl1–2), along with samples from the original Bog and AD communities used to inoculate the small reactors. Rarefaction curves of communities obtained from the small reactors were constructed using Mothur v1.44 [3] (Figure. S2). Small reactor communities showed a general leveling off, whereas diversity for the two inocula, especially the Bog inoculum, continued to climb. Paired t-tests of chemical parameters were conducted in Microsoft Excel 2016 to determine significant differences in the data obtained for the reactors in the six weeks preceding the biomass collection for sequencing (Table S3). For COD removal rates, significant differences were obtained for AD1–4 reactors with all other reactors except for AD3 vs. Bog1 and AD1 vs. Ad4. Significant differences in gas volume were found for Ctl2 vs. Bog1 and AD1–4. Ctl2 was also different than all other reactors for pH and methane. For effluent VFAs, AD4 was different than all other reactors.

A.

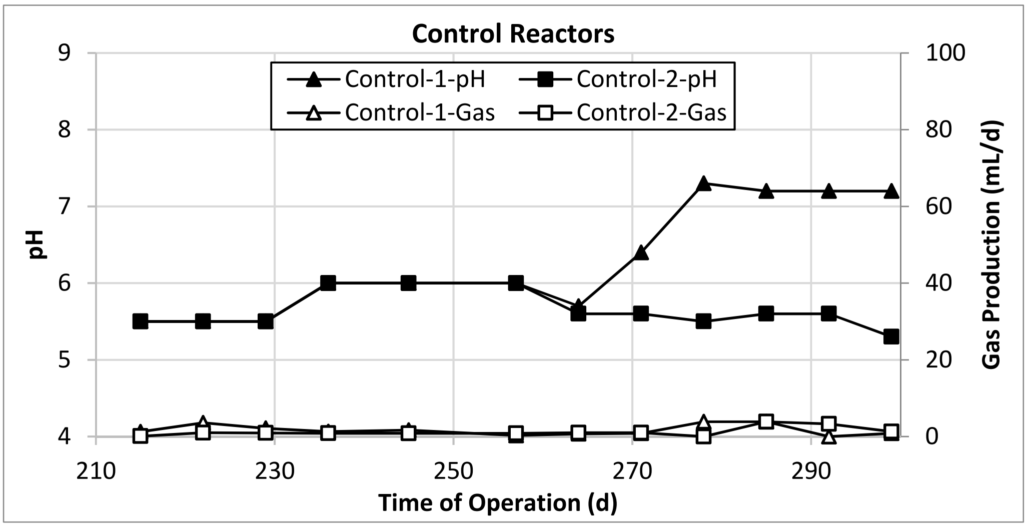

B.

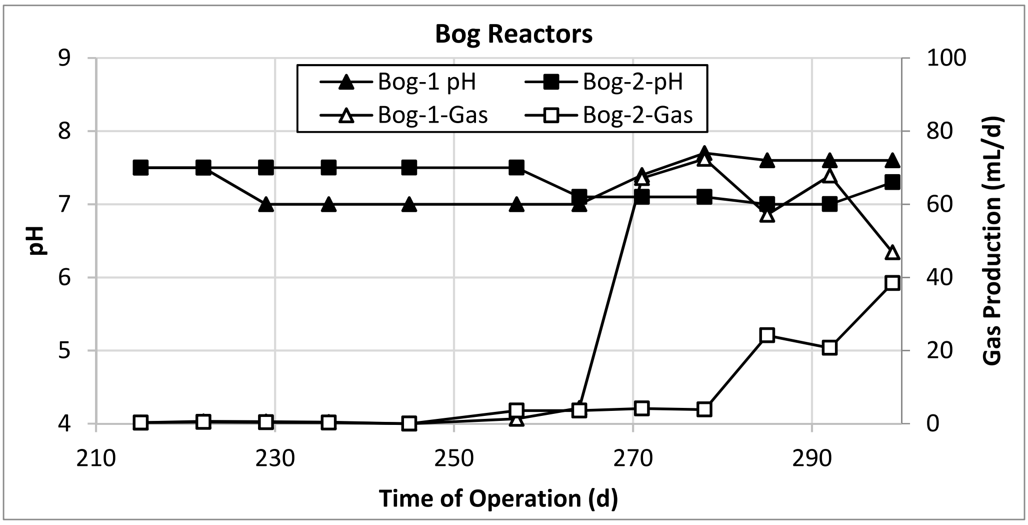

C.

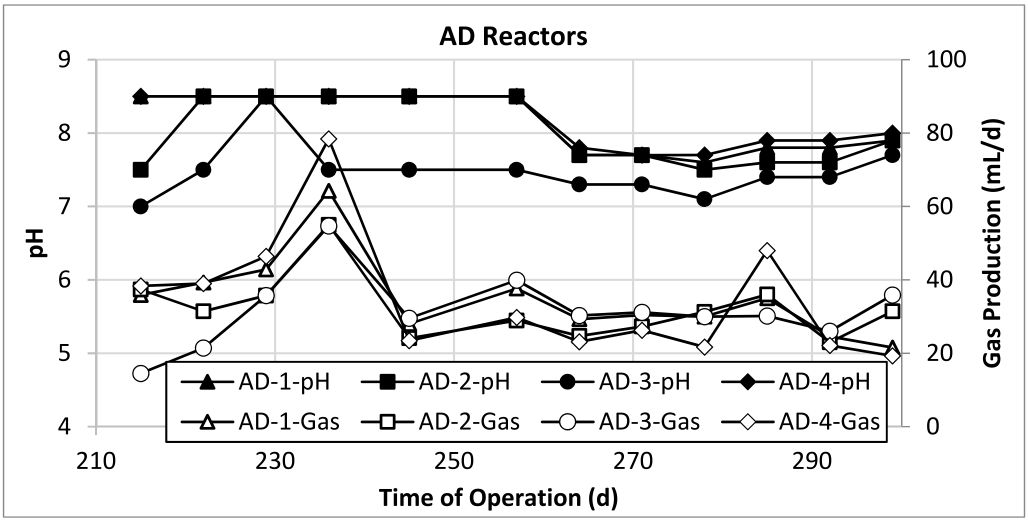

Figure S1. Performance of small anaerobic reactors over a period of 140 days prior to sampling for DNA sequencing. Reactor effluent pH and evolved gas were measured once per week.

Table S2. Carbon balance for small reactors showing consumed carbon and recovered carbon. Dissolved organic carbon was measured via Chemetrics HR+ Chemical Oxygen Demand (COD) with a measurement range of 0–15,000 ppm, and normalized to a COD standard (Chemetrics, Midland, VA). Gas volume was measured daily via syringe. Gas composition was determined via gas chromatography with a model 8610C GC (SRI Instruments, Torrance, CA) equipped with a 6-foot (183 cm) MS13X molesieve column (Restek, Bellefonte, PA) and externally calibrated with laboratory grade gases of CH_4, H_2, N_2, and CO_2. Total dissolved inorganic carbon was calculated using Henry's Law and dissociation constants for carbonic acid and bicarbonate accompanied by measurements of headspace CO_2 concentrations and pH.

Reactor	Total C Consumed (moles)	Recovered C					Fraction C Recovered
		Effluent COD (moles)	C from CH_4 (moles)	C from CO_2 (moles)	Inorganic C (moles)	Total (moles)	
Ctl1	0.56	0.71	2.8×10^{-6}	4.8×10^{-5}	0.07	0.78	1.38
Ctl2	0.47	0.77	2.9×10^{-6}	3.8×10^{-5}	0.02	0.79	1.69
Bog1	0.88	0.58	5.5×10^{-4}	1.9×10^{-3}	0.08	0.67	0.76
Bog2	0.51	0.77	1.3×10^{-4}	5.1×10^{-4}	0.16	0.93	1.81
AD1	1.52	0.15	3.2×10^{-4}	8.4×10^{-4}	0.75	0.9	0.59
AD2	1.25	0.33	3.6×10^{-4}	8.6×10^{-4}	0.58	0.91	0.73
AD3	1.00	0.46	3.1×10^{-4}	9.5×10^{-4}	0.32	0.78	0.78
AD4	1.48	0.19	3.2×10^{-4}	8.1×10^{-4}	0.74	0.89	0.63

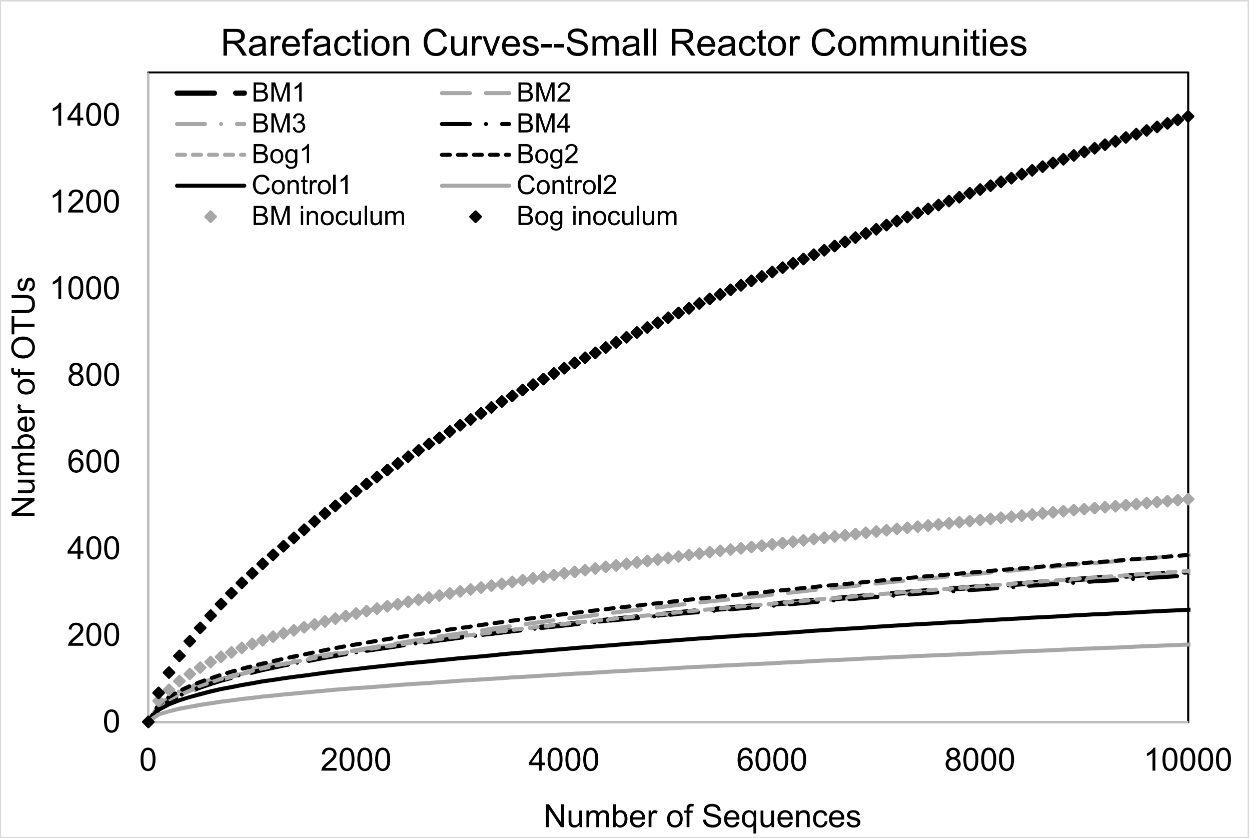

Figure S2. Rarefaction curves for microbial communities from small reactors. A total of three communities (top, bottom, and effluent) were sequenced from each reactor. Community sequences from each reactor were pooled prior to creating rarefaction curves.

Table S3. Paired t-tests of chemical parameters for small anaerobic reactors. Tests were conducted on data points collected in the six weeks prior to collection of biomass for DNA extraction and sequencing. Tests with significance values of $p < 0.005$ are marked with (*). Reactors were inoculated from an acidic bog (Bog1–2), from a large lab-scale anaerobic digester (AD1–4) or were uninoculated (Ctl1–2). Parameters tested for significance between paired reactors include, (**A**) effluent COD, (**B**) pH, (**C**) daily gas volume (mL/d), (**D**) % methane of biogas, and (**E**) effluent VFAs (mM).

(A)

	Ctl1	Ctl2	Bog1	Bog2	AD1	AD2	AD3	AD4
**Ctl1**					*	*	*	*
**Ctl2**					*	*	*	*
**Bog1**					*	*		*
**Bog2**					*	*	*	*
**AD1**						*	*	
**AD2**							*	*
**AD3**								*
**AD4**								

(B)

	Ctl1	Ctl2	Bog1	Bog2	AD1	AD2	AD3	AD4
**Ctl1**								
**Ctl2**			*	*	*	*	*	*
**Bog1**								
**Bog2**					*	*		*
**AD1**							*	*
**AD2**							*	
**AD3**								*
**AD4**								

(C)

	Ctl1	Ctl2	Bog1	Bog2	AD1	AD2	AD3	AD4
**Ctl1**			*		*	*	*	*
**Ctl2**			*		*	*	*	*
**Bog1**								
**Bog2**								
**AD1**								
**AD2**								
**AD3**								
**AD4**								

(D)

	Ctl1	Ctl2	Bog1	Bog2	AD1	AD2	AD3	AD4
**Ctl1**			*	*	*	*	*	*
**Ctl2**			*	*	*	*	*	*
**Bog1**								
**Bog2**								
**AD1**								
**AD2**								
**AD3**								
**AD4**								

(E)

	Ctl1	Ctl2	Bog1	Bog2	AD1	AD2	AD3	AD4
Ctl1		*			*	*	*	*
Ctl2			*	*	*	*	*	*
Bog1					*	*		*
Bog2					*	*	*	*
AD1								*
AD2								*
AD3								*
AD4								

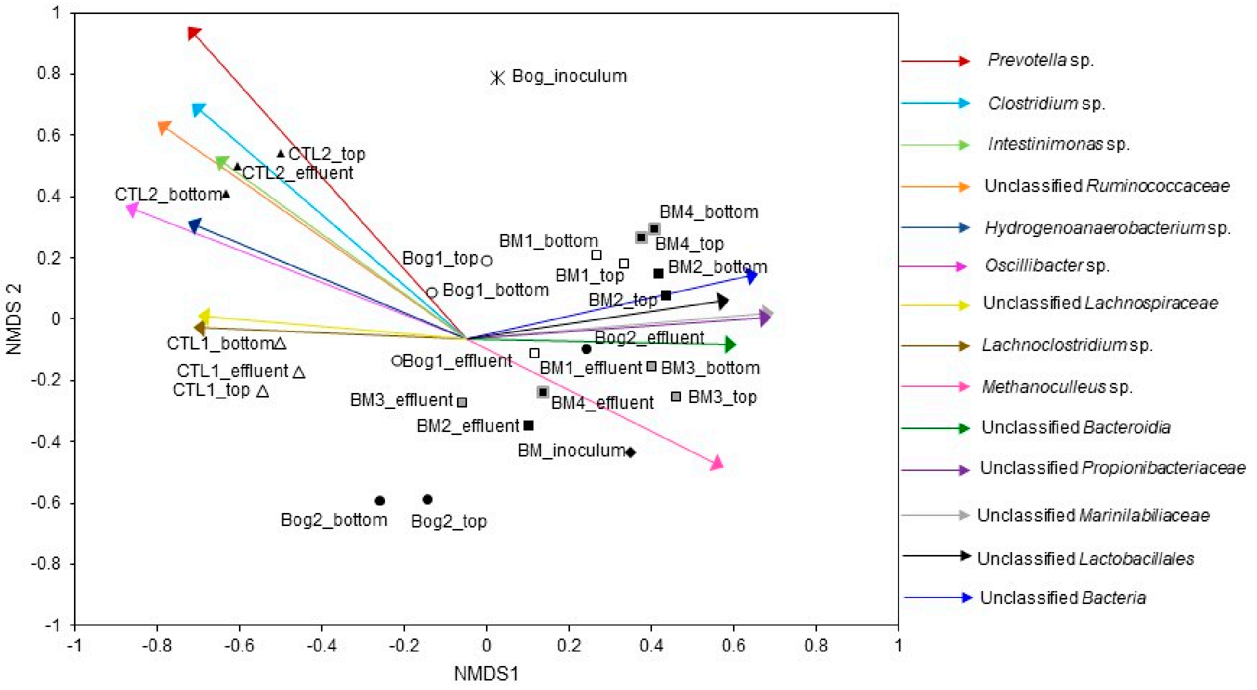

Figure S3. NMDS ordination of the matrix of Bray–Curtis distances of small reactor microbial communities as calculated in Mothur v1.44.3 [3]. Genera with p-values below 0.0001 are overlain to show their influence on ordination.

Bibliography

1. Steinberg, L.M.; Kronyak, R.E.; House, C.H. Coupling of Anaerobic Waste Treatment to Produce Protein- and Lipid-Rich Bacterial Biomass. *Life Sci. Space Res.* **2017**, *15*, 32–42. https://doi.org/10.1016/j.lssr.2017.07.006.
2. Rhodes, M.E.; Fitz-Gibbon, S.T.; Oren, A.; House, C.H. Amino Acid Signatures of Salinity on an Environmental Scale with a Focus on the Dead Sea. *Environ. Microbiol.* **2010**, *12*, 2613–2623. https://doi.org/10.1111/j.1462-2920.2010.02232.x.
3. Schloss, P.D.; Westcott, S.L.; Ryabin, T.; Hall, J.R.; Hartmann, M.; Hollister, E.B.; Lesniewski, R.A.; Oakley, B.B.; Parks, D.H.; Robinson, C.J.; et al. Introducing Mothur: Open-Source, Platform-Independent, Community-Supported Software for Describing and Comparing Microbial Communities. *Appl. Environ. Microbiol.* **2009**, *75*, 7537–7541. https://doi.org/10.1128/AEM.01541-09.
4. NIST Carbon Dioxide Available online: https://webbook.nist.gov/cgi/cbook.cgi?ID=C124389&Mask=10 (accessed on 15 October 2021).
